# UVC-PURGE: A Novel Cost-Effective Disinfection Robot for Combating COVID-19 Pandemic

**DOI:** 10.1109/ACCESS.2022.3163243

**Published:** 2022-03-30

**Authors:** Akib Zaman, Mohammad Shahjahan Majib, Shoeb Ahmed Tanjim, Shah Md. Ahasan Siddique, Shafayetul Islam, Md Shadman Aadeeb, Nafiz Imtiaz Khan, Riasat Haque, Md Rashid Ul Islam, M. Rayhan Ferdous Faisal, Siddharth Malik, Muhammad Nazrul Islam

**Affiliations:** Department of Computer Science and EngineeringMilitary Institute of Science and Technology (MIST), Mirpur Cantonment Dhaka 1216 Bangladesh; Department of Computer Science and EngineeringUnited International University, United City 130062 Dhaka 1212 Bangladesh; Department of Mechanical EngineeringMilitary Institute of Science and Technology (MIST), Mirpur Cantonment Dhaka 1216 Bangladesh

**Keywords:** COVID-19, UVC robot, medical robotics, infection control, disinfection methods

## Abstract

During the COVID-19 pandemic, surface disinfection using prevailing chemical disinfection methods had several limitations. Due to cost-inefficiency and the inability to disinfect shaded places, static UVC lamps cannot address these limitations properly. Moreover, the average market price of the prevailing UVC robots is huge, approximately 55,165 USD. In this research firstly, a requirement elicitation study was conducted using a semi-structured interview approach to reveal the requirements to develop a cost-effective UVC robot. Secondly, a semi-autonomous robot named UVC-PURGE was developed based on the revealed requirements. Thirdly, a two-phased evaluation study was undertaken to validate the effectiveness of UVC-PURGE to inactivate the SARS-CoV-2 virus and the capability of semi-autonomous navigation in the first phase and to evaluate the usability of the system through a hybrid approach of SUPR-Q forms and subjective evaluation of the user feedback in the second phase. Pre-treatment swab testing revealed the presence of both Gram-positive and Gram-Negative bacteria at 17 out of 20 test surfaces in the conducted tests. After the UVC irradiation of the robot, the microbial load was detected in only 2 (1D and 1H) out of 17 test surfaces with significant reductions (95.33% in 1D and 90.9% in 1H) of microbial load. Moreover, the usability evaluation yields an above-average SUPR-Q score of 81.91% with significant scores in all the criteria (usability, trust, loyalty, and appearance) and the number of positive themes from the subjective evaluation using thematic analysis is twice the number of negative themes. Additionally, compared with the prevailing UVC disinfection robots in the market, UVC-PURGE is cost-effective with a price of less than 800 USD. Moreover, small form factor along with the real time camera feedback in the developed system helps the user to navigate in congested places easily. The developed robot can be used in any indoor environment in this prevailing pandemic situation and it can also provide cost-effective disinfection in medical facilities against the long-term residual effect of COVID-19 in the post-pandemic era.

## I. Introduction

Since late 2019, people are facing the COVID-19 pandemic as one of the worst possible pandemics in world history. As of September 30, 2021, the total number of infected cases has risen to more than 230 million, and more than 4.5 million people have embraced death [1]. Transmission of the SARS-CoV-2 virus can occur by close physical contact, respiratory droplets of infected people and indirect contact with surfaces in the immediate environment or with objects used by an infected person [2]. Additionally, airborne transmission is possible during aerosol-generating medical procedures [3]. While being infected through physical contact and respiratory droplets can be reduced by being aware and cautious about the situation, infection through contact with infected surfaces is more difficult to handle. This difficulty occurs as it requires extra effort and resources for repeated and effective surface disinfection.

Transmission of COVID-19 through contaminated surfaces can happen both in healthcare and non-healthcare settings. Potential surfaces in health-care settings include furniture and other fixed items inside and outside of patient wards and bathrooms, for example, tables, chairs, walls, door handles, light switches and computer peripherals, electronic equipment, sinks, toilets, etc. Furthermore, the surfaces of non-critical medical equipment, such as blood pressure cuffs, stethoscopes, wheelchairs, incubators, etc. are prone to contamination [4]. These surfaces are more likely to be contaminated with the COVID-19 virus where certain medical procedures are performed concerning the suspected or infected patients. Therefore, these surfaces, especially where patients with COVID-19 are being cared for, must be properly cleaned and disinfected to prevent further transmission. Likewise, this advice applies to alternative settings for the isolation of persons with COVID-19 experiencing uncomplicated and mild illness [5]. In non-healthcare settings, sinks and toilets, electronic devices (touch screens and controls), furniture, and other fixed items, such as countertops, stairway rails, door handles, floors, and walls can be identified as the potential surfaces for spreading the virus. Transmission of the COVID-19 virus has been linked with close contact between individuals within closed settings, such as households, health facilities, assisted living and residential institution environments. In addition, community settings outside of healthcare settings have been found vulnerable to COVID-19 transmission events including publicly accessible buildings, faith-based community centres, markets, transportation, and business settings [6]. Thereby, throughout the pandemic, topmost health organizations like World Health Organisation (WHO), Centers for Disease Control and Prevention (CDC), National Health Service(NHS), etc. have constantly emphasized the necessity of safe and effective surface disinfection methods to prevent the spread of the pandemic both in healthcare and non-healthcare settings [7].

Also, Doremalen *et al.* [8] showed that the SARS-CoV-2 virus is detectable on hard surfaces up to 72 hours whereas the duration is more than 3 hours in the case of air. The List N provided by CDC [9] for disinfectants like Quaternary ammonium, Hydrogen peroxide; Per-oxyacetic acid (Per-acetic acid), Isopropanol (Isopropyl alcohol) and Phenolic are being commonly used to disinfect surfaces in medical instalments, household, office, etc. However, the disinfection of surfaces through conventional chemical disinfection methods is quite an ineffective and resource-exhausting process in a pandemic situation of this magnitude. Additionally, increasing mental health issues have been identified among the cleaning personnel as they have been involving themselves in these disinfection processes while being in very close contact with the virus [10]–[13]. In contrast, Ultraviolet Germicidal Irradiation (UVGI) is a non-touch disinfection method that uses short-wavelength Ultraviolet-C (UVC) light to kill or inactivate microorganisms by destroying nucleic acids and disrupting their DNA, leaving them unable to perform vital cellular functions [14]. Currently, in a few of the medical installations, static UVC lights are being used to disinfect the medical equipment and in some cases the whole room. However, this cannot be used to properly disinfect the whole room especially the congested spaces of the room which leaves a significant amount of shaded places contaminated. Moreover, the procedure is very costly. Again, there has been a significant amount of research [15]–[21] recently about the required amount of UVC irradiation for the inactivation of SARS-CoV-2 (coronavirus). The research findings of these studies can be utilized by developing a mobile robotic platform to address the limitations of the static UVC disinfection method. Some of the UVC disinfection robots [22]–[34] are available in the market. However, with an average market price of 55,165 USD, these existing robots are not cost-effective which provides a scope of improvement in cost-effectiveness and functionality.

**FIGURE 1.**
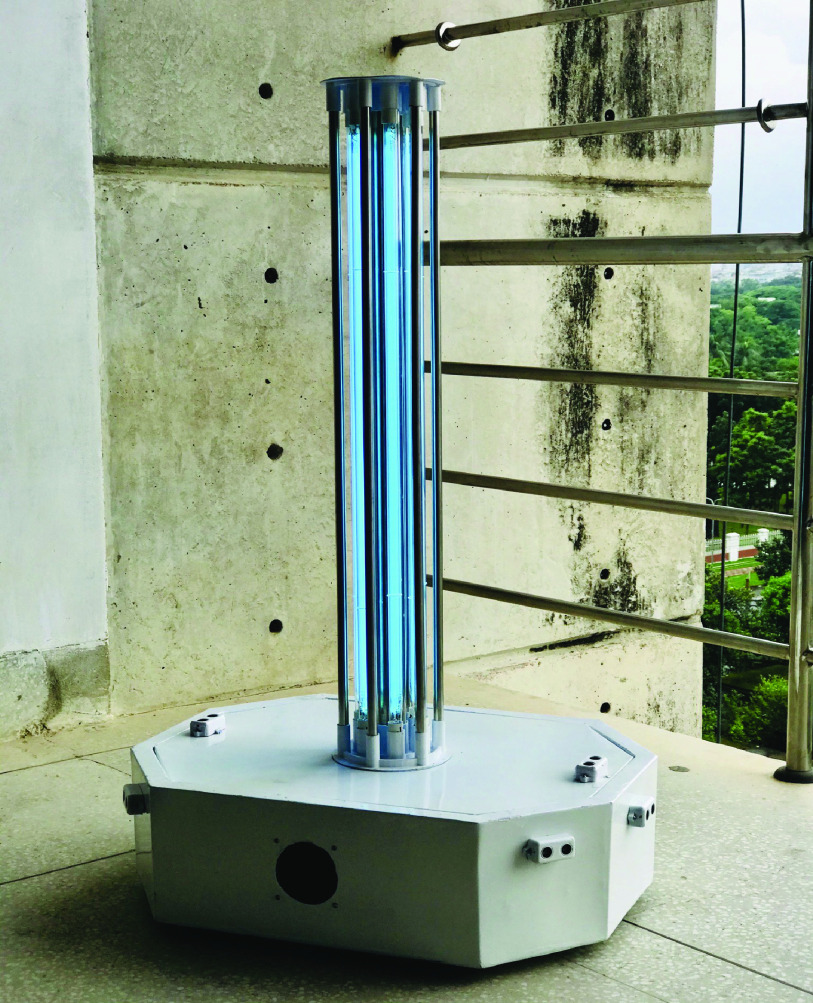
UVC-PURGE; Champion in the application criteria of the UK medical robotics challenge for contagious disease 2020.

Thus, the objective of this research was to develop a cost-effective UVC robotic system to inactivate SARS-CoV-2 (coronavirus) for providing safe, time-efficient, and cost-effective disinfection for people from all types of socioeconomic backgrounds. To attain this objective, a semi-autonomous robotic system named UVC-PURGE (Figure 1) was developed and the performance of the robot in terms of inactivating microorganisms (similar or more resistant than SARS-CoV-2) and autonomy of the robot in a practical environment was evaluated. Additionally, the usability of the developed system was evaluated through a hybrid approach of Standardized User Experience Percentile Rank Questionnaire (SUPR-Q) [35] forms and subjective evaluation. Moreover, with a price of less than 800 USD, UVC-PURGE is likely to be the most cost-effective UVC robot compared with the similar robots prevailing in the market. In this current pandemic situation, the developed robot can be used in any indoor environment such as COVID patient ward, ICU, operation theatre, office, classroom, corridor, personal apartment, etc. Moreover, the robot can serve as a cost-effective Infection Prevention and Control (IPC) tool in medical facilities and biomedical laboratories against the long-term residual effect of COVID-19 [36], [37] in the post-pandemic era. One of the functional units of UVC-PURGE is being used by a reputed telecommunication monitoring company of Bangladesh as a pilot user. Moreover, this robotic system has been awarded as the best robotic system in the Application criteria of the UK Medical robotics challenge for contagious disease 2020 [38], [39] organized by the UK Robotics & Autonomous System (UK-RAS) network.

This paper is divided into six sections. Section 2 describes the background of this research by highlighting limitations of the current disinfection methods, prior works related to UVC disinfection, a comparative market analysis of prevailing UVC robots, and studies related to the lethal dose of UVC for SARS-CoV-2 (coronavirus). In section 3 the requirement elicitation process has been elaborately described. The design and development of the robotic system have been discussed in section 4. Furthermore, the evaluation of the developed system is described in Section 5. Finally, Section 6 summarizes the contribution of this research along with the limitations and the future scopes.

## II. Background

### A. Existing Disinfection Methods

United States Environmental Protection Agency (EPA) suggests that all products on List-N can kill the SARS-CoV-2 when used according to the label directions [40]. List-N [9] has been referred by CDC where the disinfectants have been suggested based on a few categories such as a) tested against SARS-CoV-2; b) kills a human coronavirus similar to SARS-CoV-2 and c) kills a harder-to-kill pathogen than SARS-CoV-2. Some of the common chemical disinfectants against SARS-CoV-2 are Quaternary ammonium, Hydrogen peroxide; Peroxyacetic acid (Peracetic acid), Isopropanol (Isopropyl alcohol) and Phenolic. Moreover, some of the prevailing surface disinfectants all over the world such as Sodium hypochlorite (bleach/chlorine) at a recommended concentration of 0.1% or 1,000ppm, Alcohol at 70-90%, etc has been mentioned by WHO [7]. Generally in indoor spaces, routine application of disinfectants to surfaces via spraying is not recommended for disinfection of COVID-19, rather a cloth or wipe soaked in the disinfectant has been recommended [7].

However, this chemical disinfection method poses some formidable disadvantages. One of the major disadvantages is the threat of handling toxic/corrosive chemicals during storage and disinfection procedures. Disinfectants containing chlorine can produce skin or mucous membrane irritation upon prolonged contact. Similarly, corrosion when applied to metals and possible co-carcinogenic properties may eventually lead to skin cancer. On a similar note, these disinfectants are not suitable for use with various materials such as curtains, cushions, bedsheets, etc as the longevity of these materials can be damaged. Additionally, disinfectants with alcohols are extremely volatile and flammable which can create biohazards due to the longer duration of storage [41]. Again, Hypochlorite is rapidly inactivated in the presence of organic material. Thus, it is important to clean the surfaces thoroughly with soap and water or detergent using mechanical action such as scrubbing or friction before applying the disinfectant. This technique is time-consuming and inefficient in the present pandemic situation where the hospitals are being overwhelmed with a huge number of patients. In addition, chemical disinfectants using chlorine solutions need to be prepared every day and are needed to be stored in opaque containers in a well-ventilated and covered area that is not exposed to direct sunlight. High concentrations of chlorine can lead to side effects for vulnerable people such as people with asthma [6]. The chances of creating the wrong mixture of chemical disinfectants can also be very catastrophic [42]. Additionally, while carrying out the disinfection process in various infrastructures, the maintenance staff are recommended to follow strict control protocols and use gowns, gloves, protective eyewear, and masks. However, performing disinfection in large spaces for a prolonged period can be physically exhausting. In addition, there are some other constraining factors like the evacuation of the room beforehand, movement of people, inadequate supply of the safety equipment, etc. Moreover, during the disinfection process, a cleaner needs to work in close contact with the virus, which leaves a significant adverse effect on their mental health [10]–[13].

### B. UVC Disinfection Method Against SARS-CoV-2

The United States Environment Protection Agency (EPA) [43] divided the viruses into three subgroups based on their size and type. These are (i) enveloped viruses (such as coronavirus, easiest to inactivate); (ii) large (50-100nm) non-enveloped viruses (such as adenovirus and rotavirus, harder to inactivate than enveloped viruses); and (iii) small (less than 50nm) non-enveloped viruses (most difficult to inactivate, such as rhinovirus). The responsible pathogen, SARS-CoV-2, is a large, enveloped, single-stranded RNA virus and a member of the family Coronaviridae, the order Nidovirales, and the genus Coronavirus. They have a spheroid shape with a diameter of about 100–150 nm, are covered with spike proteins on the outside and have an RNA strand length of 27–32 kb on the inside [44]. There are four genera of this virus known as alpha, beta, delta and gamma among which alpha and beta CoVs are known to infect humans [45]. So far, seven coronaviruses have been found to infect humans and cause respiratory diseases. Four of these seven coronaviruses are known to be the causative agents of common colds (HCoV-229E, HCoV-NL63, HCoV-OC43 and HCoV-HKU1), which usually only result in milder infections, but the coronaviruses MERS-CoV (MERS: Middle East Respiratory Syndrome), SARS-CoV and SARS-CoV-2 have claimed many lives [46]. Ultraviolet light at wavelengths between 200 and 280 nanometers, also known as UVC light is a long-established disinfectant in health care settings. Over the last 10 years, hospitals around the world have adopted machines that sterilize rooms and equipment with powerful blasts of UVC light. UVC radiation kills or inactivates microbes by damaging their Deoxyribonucleic Acid (DNA). The principal mode of inactivation occurs when the absorption of a photon forms pyrimidine dimers between adjacent thymine bases and renders the microbe incapable of replicating. UVC can be used to disinfect the air, water, and surfaces, although micro shadows and absorptive protective layers limit surface disinfection [47]. Beckett [48] mentioned the mechanism of UVC while developing a robotic platform named “VIOLET”. According to him, UVC causes DNA to change shape and acts like molecular scissors to cut the genetic material and causes little cuts in it. Complex organisms and even some bacteria can repair those small lacerations themselves. However, viruses being molecularly much simpler than bacteria do not stand a chance against UVC irradiation.

Unlike the disinfection methods described in the previous subsections disinfection methods using a UVC lamp are more effective and safer. UV disinfection is a chemical-free process and leaves behind no residue. Additionally, it requires no transportation and storage, or handling of toxic or corrosive chemicals. Disinfection without human intervention (such as preparation of disincentive agents, maintenance of the reagents, etc.) eliminates the probability of error during the disinfection process. However, longer and close exposure UVC(254nm) has a direct impact on human tissue. Large amounts of UVC radiation is recognized to be a potential human carcinogen. Temporary eye and skin damage, such as cornea injury, has been observed in rare cases of prolonged direct UVC light exposure. An alternative way might be to set static UVC lamps inside the room and disinfect the room in absence of humans. Furthermore, it is not cost-efficient to install UVC light in each room of any infrastructure such as hospital, office, etc., especially in the perspective of developing and under-developed countries. Even if it is managed within the budget, this process might leave out many shaded places, which will remain contaminated.

### C. Prevailing UVC Disinfection Robots: a Market Analysis

A market analysis was conducted to find out the prevailing robots in the market for UVC disinfection as shown in Table 1. It has emerged from the analysis that most of the robots are not constructed considering the small form factor ( $ < 55 \times 55$ cm footprint [14]) which is not suitable for navigating in congested places. Real-time video feed has not been integrated into most of the prevailing robots. A variety of controllers have been observed in the existing robots such as mobile applications, separate controller devices, desktop apps, etc. Moreover, the average market price of the prevailing robots is 55,165 USD ranging from a minimum price of 10,000 USD to a maximum value of 1,25,000 USD. The COVID-19 pandemic has affected people from all socioeconomic backgrounds and left them with an immediate need for a cost-efficient disinfection system. Considering the income range of people from low and middle socioeconomic backgrounds, the average price of prevailing robots is very expensive.

**TABLE 1:** Prevailing UVC Disinfection Robots

REF Price	Name	Form Factor ( $\text{L}\times \text{W}\times \text{H}$ cm)	weight (kg)	Small Form Factor	Real-time Video Feedback	Controller	Autonomous
[22], [23] $\$ $81,000	Xenex(2014)	$76\times 51 \times 97$	NA	No	No	Separate Controller	No
[24] $\$ $12,700	SIFROBOT 6.57(2019)	$41\times 41 \times 135$	33	Yes	No	Not Available	Yes
[25] $\$ $17,950	SIFROBOT 6.53 (2019)	$63.5\times 48 \times 153$	45	No	Yes	Not Available	Yes
[26] $\$ $10,000	Su-01 (2020)	$50\times 55 \times 139$	75	Yes	No	Windows App	Yes
[27] $\$ $25,000	Helios (2020)	$64\times 54 \times 173$	NA	No	No	Not Available	Yes
[23], [28] $\$ $1,25,000	TruD (2020)	NA $\times $NA $\times 168$	NA	No	No	Not Available	No
[29] $\$ $90,000	ZENZOE (2020)	$74\times 66 \times 197$	27	No	No	Mobile App	Yes
[30] $\$ $80,000	SEIT-UV (2020)	$89\times 65\times 29.7$	120	No	No	Mobile App	Yes
[31], [32] $\$ $70,000	HERO21 (2020)	$70\times 55 \times 155$	NA	No	Yes	Mobile App	Yes
[33], [34] $\$ $40,000	Adibot-A (2021)	$60\times 60 \times 169$	100	No	No	Mobile App	Yes

### D. Required Lethal Dose for the Inactivation of SARS-COV-2

There has been a significant amount of research (Table 2) recently about the required amount of irradiation for the inactivation of SARS-CoV-2. Most of the studies have stated that the lethal dose for inactivation of SARS-CoV-2 ranges from rational (LD90 i.e 90%-LD99.9 i.e 99% of disinfection) to complete (LD 99.999 i.e. 99.999% of disinfection). The values in Table 2 have been expressed in terms of $mJ sec/cm^{2}$.

**TABLE 2:** Literature Regarding the Lethal Dose of SARS-CoV-2

Reference	Complete Lethal Dose (LD99.999) in $mJ sec/cm^{2}$	Rational Lethal Dose (LD99.9) in $mJ sec/cm^{2}$	Rational Lethal Dose (LD90) in $mJ sec/cm^{2}$
[15]	–	–	3.7
[16]	2.19	–	1.6
[17]	1.95	–	0.54
[18]	–	–	0.32 ± 0.07
[19]	11.25	3.75	–
[20]	16.9	3.7	–
[21]	–	–	2.7

The studied works suggested that the rational average value of the lethal dose (D90 D99) ranges from $0.32~mJ sec/cm^{2}$ to $3.75~mJ sec/cm^{2}$. However, a total inactivation would require a UVC dose range from $1.95~mJ sec/cm^{2}$ to $16.9~mJ sec/cm^{2}$.

### E. Findings From the Background Study

This background study reveals some significant limitations to address and some interesting opportunities to work for. Firstly, analyzing the prevailing chemical disinfection methods, many drawbacks have been found such as a) handling of toxic/corrosive chemicals during storage and disinfection procedures; b) flammability and probability of bio-hazard; c) physical exhaustion of the maintenance staffs as well as mental fear of remaining in close contact with the virus; d) probability of creating a wrong disinfectant mixture and e) resource exhaustive process of daily regeneration and storage. Secondly, setting static UVC lamps may address the limitations observed in the chemical disinfection method. However, cost-inefficiency is a big concern in this method. Additionally, it leaves out a lot of shaded places, which remain contaminated. Thus, the necessity for a mobile robotic system with UVC lights can be perceived. Thirdly, a market analysis was conducted to analyze the prevailing UV robots in the market. The average market price of the prevailing robots is 55,165 USD which is huge considering the pandemic situation, which has affected the whole world including people from all types of socioeconomic backgrounds. Thus, there is substantial scope for increasing the cost efficiency of a UVC robot. Moreover, analysis of the current features of the prevailing robots shows that a small form factor ( $ < 55 \times 55$ cm footprint) is needed to be considered while developing a UVC robot to ensure the capability of moving in a congested place. Real-time video feedback to the users is also important for ensuring a better user experience during controlling the robot. Finally, recent studies related to the lethal dose for the inactivation of SARS-CoV-2 (coronavirus) suggested that the rational lethal dose (LD90-LD99.9) ranges from 0.32 $mJ sec/cm^{2}$ to $3.75~mJ sec/cm^{2}$. However, a total inactivation would require a UVC dose range from $10.6~mJ sec/cm^{2}$ to $16.9~mJ sec/cm^{2}$, which is not energy-saving and safe for human use. The findings from this background study have been used to construct the research framework. Therefore, in this study, we have focused to develop a cost-effective UVC robotic system capable of nullifying the disadvantages of both chemical and static UVC disinfection methods. At the same time, the proposed system should effectively produce a sufficient level of UVC irradiation to inactivate the SARS-CoV-2 virus. The upper limit of rational lethal dose $3.75~mJ sec/cm^{2}$ has been chosen to be implemented in the robotic system.

## III. Requirements Elicitation

The objective of the requirements elicitation study was to understand the context of use, specify requirements of the concerned stakeholders and identify the possible challenges in developing a sustainable and cost-effective robotic disinfection system against SARS-CoV-2. Research shows that failure in understanding users’ requirements properly at the initial stage of design and development usually leads to several incorrect and incomplete assumptions [49]. Based on these facts, the requirements elicitation study was conducted with healthcare and cleaning professionals from five of the renowned government hospitals of Bangladesh in the month of May 2020. In the following subsections, the profiles of the participants, the study procedure, and the results of the requirement elicitation study are presented.

### A. Participants’ Profile

For conducting the requirements study, the participants were chosen with the help of the Snowball Sampling method [50], [51]. The authors had connections with three doctors working in two different government hospitals. By using the snowball sampling method, the rest of the participants were chosen with their help. The final list of participants included 7 doctors, 6 nurses, and 16 maintenance and cleaning staff. Among the participants, all of the cleaning and maintenance staff work in all possible environments where a disinfection system can function. However, the doctors and the nurses who took part in the study don’t work directly in all possible environments (such as, disinfecting patient washrooms, etc.) They were more knowledgeable regarding the spread of germs and methods of disinfection compared to the cleaning and maintenance staff. Hence, they were included in the requirement elicitation study for disseminating their valuable insights and knowledge.

The average age of the participants was 32 years and the range of the participants’ age was between 21 to 60 years. Out of the seven doctors, three of the doctors were interned doctors who graduated in the previous year but had working experience in the COVID patient wards. The remaining three doctors had professional experiences of 10, 14, and 15 years respectively. Two of them were medicine specialists and the other was a neurosurgeon. The six nurses were graduates from different nursing colleges and their average working experience was 6.9 years. The 16 maintenance and cleaning staff had an average work experience of 3.6 years. All of them had performed duties during the pandemic situation as frontline helping hands. All the participants were familiar with different chemical solution-based disinfecting and sanitizing methods for protection against COVID-19.

### B. Study Procedure

Members among the authors visited five different hospitals in groups consisting of three members for conducting the requirements elicitation study. Each of them wore Personal Protective Equipment (PPE) as a precautionary measure considering the higher probability of getting infected with Corona Virus at the hospitals. Before visiting, appointments were made with the doctors. With the help of the doctors, the authors got the opportunity to meet with the nurses along with the cleaning and maintenance staff in the respective hospitals.

At first, the participants were asked about their different demographic information such as age, working experience, educational qualifications, etc., and a consent form was signed by each participant. The participants were ensured that their information will be used solely for research purposes and none of their personal information or images would be disclosed. The participants were also informed that based on our background study, our research mainly focused on developing a robotic system-based UVC disinfection method for improving prevailing disinfection procedures and the ongoing study is for exploring the user requirements.

An open-ended interview questionnaire was set for the participants of the study containing questions regarding the disinfection procedure of the wards and equipment, opinions about improving the system, cost regarding the disinfection procedures, etc. We enquired about which equipment was frequently used and which places in the hospitals were frequently disinfected. The participants were further asked about the chemicals and tools used for disinfection purposes. Also, information regarding the pros and cons of existing disinfection methods was probed from them. Furthermore, from the doctors, their opinions regarding the use of UV rays in disinfection were sought.

During the interviews, the participants were encouraged to talk freely and comfortably about the issues that were probed. Additional questions were asked for clarification in case of issues where doubts were created in our minds. Upon concluding the interview, the participants were asked if they would like to add any additional information which we might have missed.

All the interviews were audio-recorded with the participant’s permission. Later the audio recordings were transcribed by different members among the authors. At the time of the interviews, necessary notes were taken as well. The duration of each of the interviews was between 15 to 20 minutes. In the end, each and every measure is taken while conducting the interviews helped us to obtain several meaningful pieces of information.

### C. Results and Discussions

A very rich and meaningful interview dataset was prepared through the interview. From the interview questions, it was collected that the disinfection mandate of the hospitals was to disinfect surfaces of the COVID-19 patient ward of the suspected patient using Sodium hypochlorite Agent thoroughly. The cleaning personnel admitted their fear of being infected as they operate in the infected place to clean. One of the hospitals was using a UVC machine to disinfect the equipment. However, they claim that a mobile system will be effective for disinfecting the equipment. They will prefer a system to disinfect the whole surfaces of the rooms like COVID-Patient ward, ICU, Operation Theatre (OT), etc. In order to extract the important ideas and themes from the dataset, thematic analysis of the interview data was conducted following the Six Phase Approach for Thematic Analysis [52], [53]. The themes obtained as outcomes from the thematic analysis are presented in the following sub-subsections.

#### 1) Cost-Effective Disinfection

One of the most important concerns that were obtained from the thematic analysis was the necessity for a cost-effective disinfecting solution. This problem was cited by 57% of the doctors, 50% of the nurses, and 88% of the cleaners. Due to COVID-19, the participants highlighted the increased necessity for different cleaning equipment including sodium hypochlorite agent (71%), brooms, mops and buckets (51%), and PPE (76%). According to the doctors (42%), more cleaning and maintenance staff have been hired after establishing the corona wards and their salaries have also been increased. The participants (66.66) also mentioned other necessary costs that have increased due to COVID-19 which includes the cost of ventilators, ICU equipment, medicines, etc. Hence cutting down disinfecting costs can also help financially with the other costs as well.

#### 2) Time Efficient Disinfection

Another important theme that emerged from the thematic analysis was the necessity of a system to aid in saving and managing time for disinfection. This problem was cited by all (100%) of the cleaners and (28%) of the doctors. Due to the COVID-19 pandemic, the floors of the hospitals are being frequently cleaned and different equipment is being frequently sanitized with disinfectants. Though additional cleaning and maintenance staff had been hired, it still was not enough. A major concern regarding the disinfection method was that generally 30–40 minutes are required to wipe a whole room and almost 15–20 minutes overhead for drying that up. Moreover, for a larger hospital, this time rises to almost more than an hour. Most of the cleaner and maintenance staff (87.5%) claimed that the limited number of cleaning staff along with the increased pressure of patients has made this disinfection procedure unsustainable. Almost all the cleaners had to work overtime frequently. Furthermore, the cleaners also mentioned (75%) about being deployed to clean different rooms at different levels of the hospital building which required a lot of time. The cleaning and maintenance staff also mentioned that they required additional time for managing the inventory of cleaning tools. The increased repetitive cleaning tasks were found to be stressful (93%) by them and they were finding very few time slots to have a decent break. During the thematic analysis, it was also found that the participants (72.4%) were expecting some sort of automated robot to help with the disinfecting tasks and save time after our idea about developing a robot was mentioned to them.

#### 3) Navigation in Congested Place

The capacity to navigate in congested places was another issue that emerged during the interview. This problem was identified when the cleaning and management staff were asked about the locations they had to clean and disinfect. They mentioned different locations in the hospitals where congested work environments caused difficulty in cleaning including changing rooms used for wearing PPE (62.5%), hospital wards (18.75%), washrooms (56.25%), and laboratories (50.00%). When this issue was discussed with the other participants, they expressed similar concerns. The doctors (42.86%), as well as the nurses (33.33%), suggested that the robotic system that we were intending to develop should be able to operate in these congested areas. While discussing the design of the robotic system the participants (51.72%) mentioned that the final structure of the system should occupy less space and hence have a compact design.

#### 4) Simple Robotic Controller and User Interface

While conducting the thematic analysis, another concern that was identified was the requirement of a simple design of the controller of the robot. This issue was brought up by one of the doctors and when it was further discussed with the other participants, it was unanimously agreed upon by all of them. Most of the cleaning and maintenance staff (62.5%) had primary-level education and none of them had higher secondary or college-level education. Complex robotic controller design with several appendages and functionalities would result in redundant features which could not be operated properly by the cleaning and maintenance staff. The necessity of a simple user interface of the controller was raised by the maintenance and cleaning staff. Most of them (75%) mentioned that they were concerned about how they would be able to control a robot on their own. Furthermore, very few members of the cleaning staff (37.5%) had experience in using smartphones. But the majority of them (62.5%) had seen or operated remote-controlled toy cars. It was suggested by the doctors (57.5%) to make a mobile application whose layout is similar to a simple remote controller and an individual with little or no education can easily be trained to use it.

#### 5) Intuitive Feedback for Navigation

During the conversations, a group of doctors (42.85%) was also curious about how a user would obtain information about the position of the disinfecting robot. This topic was further discussed with all other participants as well. Since the idea of using a mobile app was already discussed, a significant portion of the participants (44.8%) were feeling that including live feedback from a camera can greatly help in providing real-time feedback to the user. Furthermore, another group of doctors (42.86%) also felt that attaching a camera with the robot would be really helpful for the user.

#### 6) Harmful Impacts of UV Rays

Although we have identified that UV rays can be used as a non-chemical disinfecting agent through literature review but all of the doctors (100%) raised concerns regarding the harmful impact of UV rays. The issues underlying their concerns include potential skin problems (100%), damage to the eyes (85.71%), loss of immunity (71.43%), etc. One of the doctors requested us to place warning messages and warning signs on the robot in case we implemented the UV ray-based robot. But despite the use of UV rays, almost all the participants (96.55%) agreed that if precautionary measures and necessary training were ensured, then the UV rays-based disinfecting robot would bring more advantages compared to the potential risks. Moreover, one of the doctors mentioned that if a user can control the robot from a safe distance or from a neighboring room that might nullify this safety issue.

### D. Requirements Elicitation Summary

As a result of conducting the requirements elicitation study, the expectations, demands, and concerns of the stakeholders have been obtained. Based on the findings from the study, the design requirements could be identified. From the themes that were found from the requirements elicitation study, it was clear that the users expected a small and compact design of the robot capable of navigating in congested places. Furthermore, the design requirements of the user interface for the system could also be comprehended for the emerging themes. The users demanded a remote-like controlling interface for controlling the robot. Moreover, the users also expected that the live video feedback will be obtained from the robot, and hence it is to be included in the controller design as well.

## IV. Design and Development of UVC-Purge

UVC-PURGE was developed with a vision to provide safe and cost-effective disinfection amidst the COVID-19 pandemic. Considering all the findings of the requirement analysis, the objective of the design procedure of the robot was to make it as effective as possible with a small form factor to move in congested spaces that can be monitored easily using real-time video feedback. Additionally, the robot should have an obstacle detection mechanism to avoid collision and must ensure UVC irradiation equivalent to the rational lethal dose of $3.75~mJ sec/cm^{2}$.

### A. Design Architecture and Considerations

**FIGURE 2.**
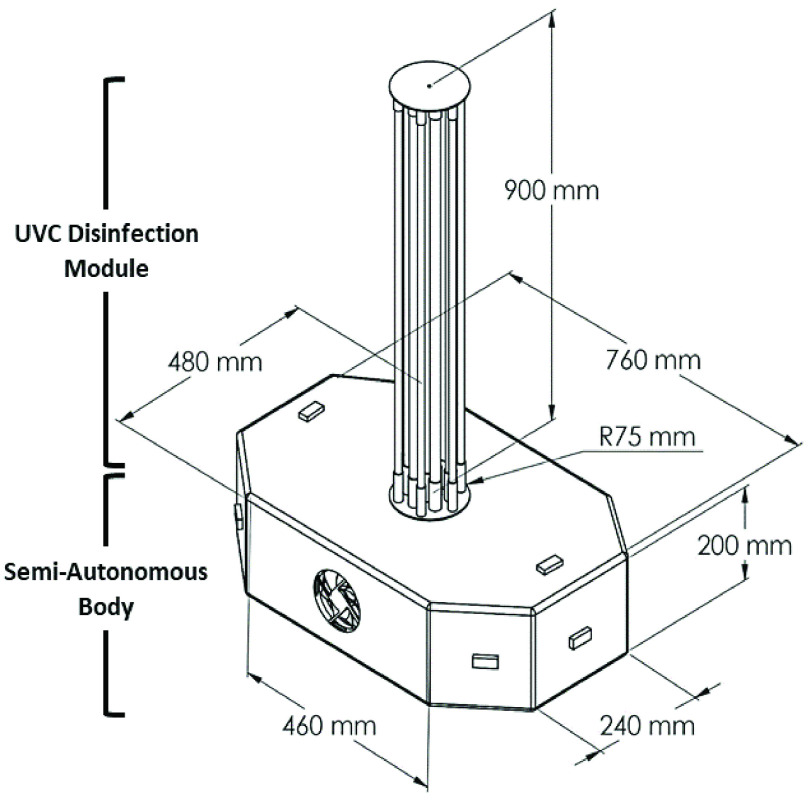
CAD model of the designed robot.

A Computer-Aided Drawing (CAD) model of the developed system is given in Figure 2 where the essential components of the robot have been highlighted. The robotic structure is made of two main parts: 1) the semi-autonomous body and 2) the UVC disinfection module. The octahedral-shaped body consists of a) Stainless Steel (SS) chassis with drive subsystem, b) processing unit, c) communication subsystem, d) navigation subsystem, and e) power subsystem. The base structure was made of mild steel for ensuring strength. The octahedral structure was considered to create a 360-degree positioning of the obstacle avoidance sensors to detect both vertical and horizontal obstacles. The UVC disinfection module has six UVC light sources (mercury type TUV PL L 35W) that can generate a peak wavelength at 254 nm when vertically mounted in a circular reflective SS enclosure with a 3D printed base. This SS enclosure increases the total output of the lamp by reflecting the UVC radiation [54] as well as serves as physical protection to the lamps. Additionally, the central positioning of the lights ensures stability and reduces jerky movements. Moreover, a small form factor was ensured by designing the whole structure within the dimension of $52\times 48\times 101$ cm (length, width, height). The total mass of the robot is approximately 25 kg.

### B. Calculation of Disinfection Time

Disinfection time for a one square meter area was taken as a unit to calculate the disinfection time. At first, The summation of radiation flux of the lamps has been calculated using Stefan Boltzmann’s (S-B) Law of radiation flux. The temperature effect on the radiative efficiency of the UVC lamps was also considered (see Appendix A: Figure 9). Then using the equation of calculating irradiance, the amount of time required for the disinfection of 1 square meter area was calculated which will ensure the rational lethal dose of 3.75 $mJ sec/cm^{2}$. Through calculation (Appendix A) it was found that by traversing at 1 meter in 5.19 seconds, UVC-PURGE can provide an effective lethal dose for that 1 m $\times $ 1 m square meter surface. Thus, the maximum speed of the robot has been set to $19.3~cm/sec$ or $0.193~m/sec$. The robot is capable of disinfecting a standard bedroom of (4 m $\times $ 5 m) with furniture within 4–5 minutes (see Appendix A: Figure 12).

**FIGURE 3.**
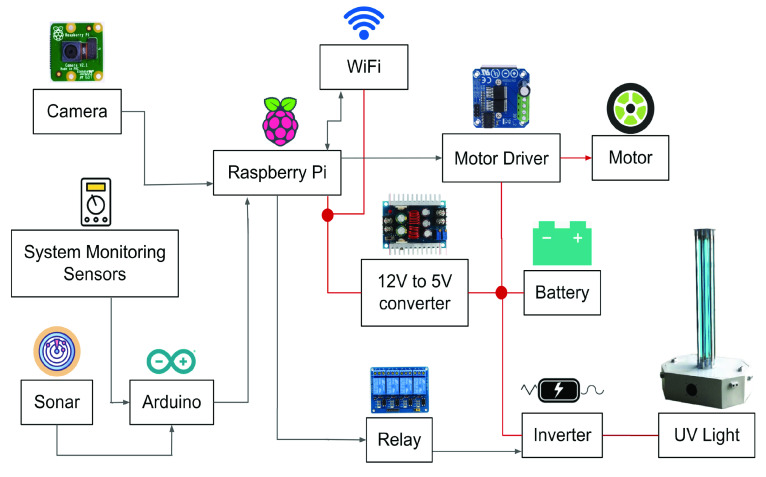
The system architecture of the robotic system.

**FIGURE 4.**
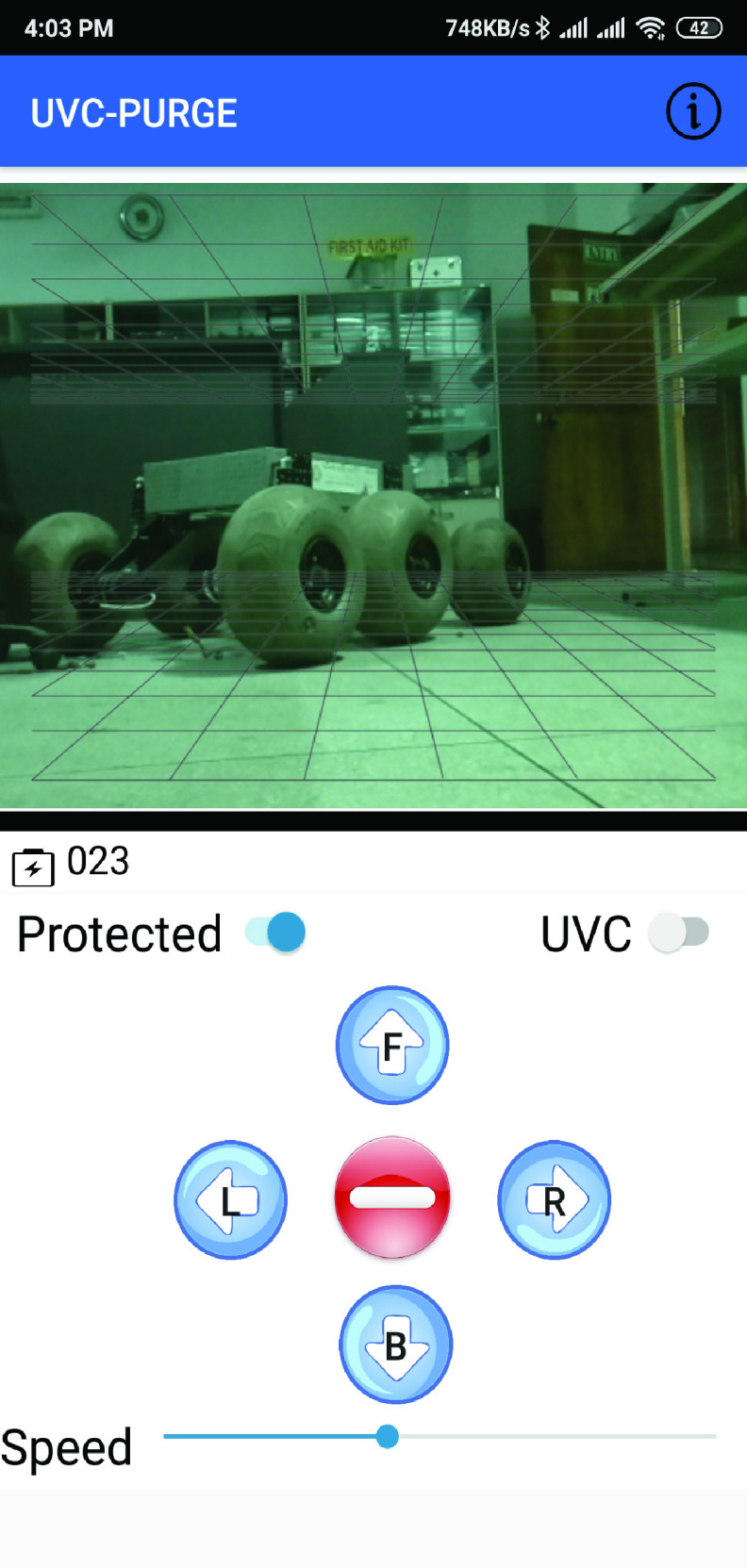
UI design of UVC-PURGE mobile application.

**FIGURE 5.**
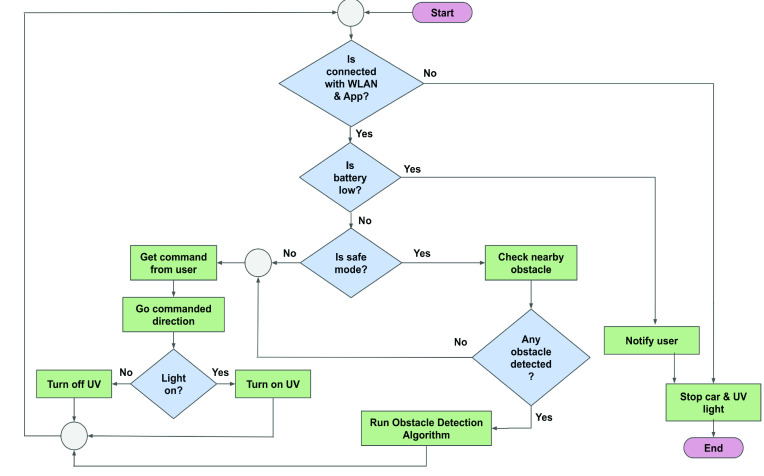
Workflow diagram of UVC-PURGE controller.

**FIGURE 6.**
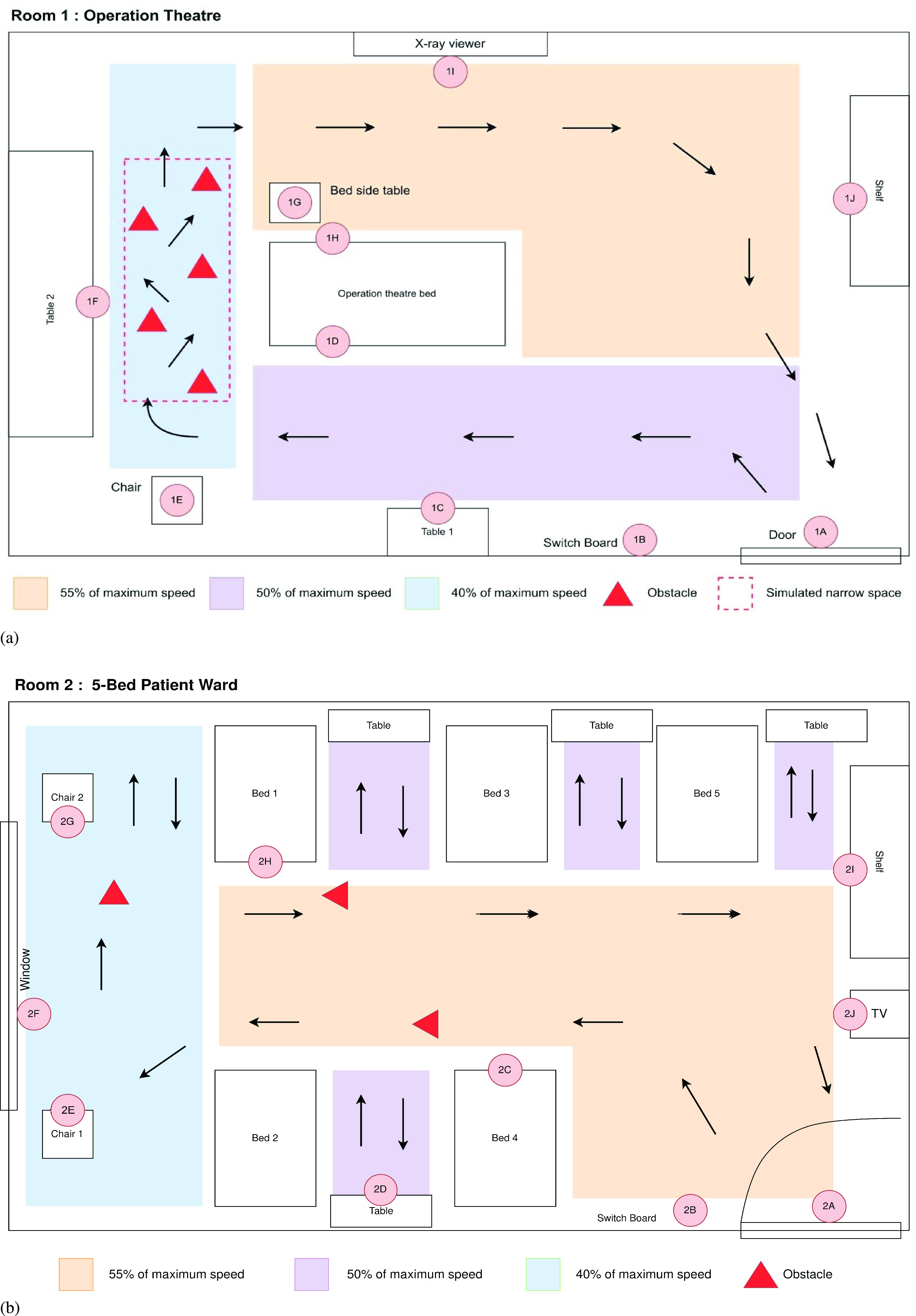
(a) Schematic diagram of the operation theatre (Room-1) of evaluation study (b) Schematic diagram of the 5-Bed patients’ ward (Room-2) of the evaluation study.

**FIGURE 7.**
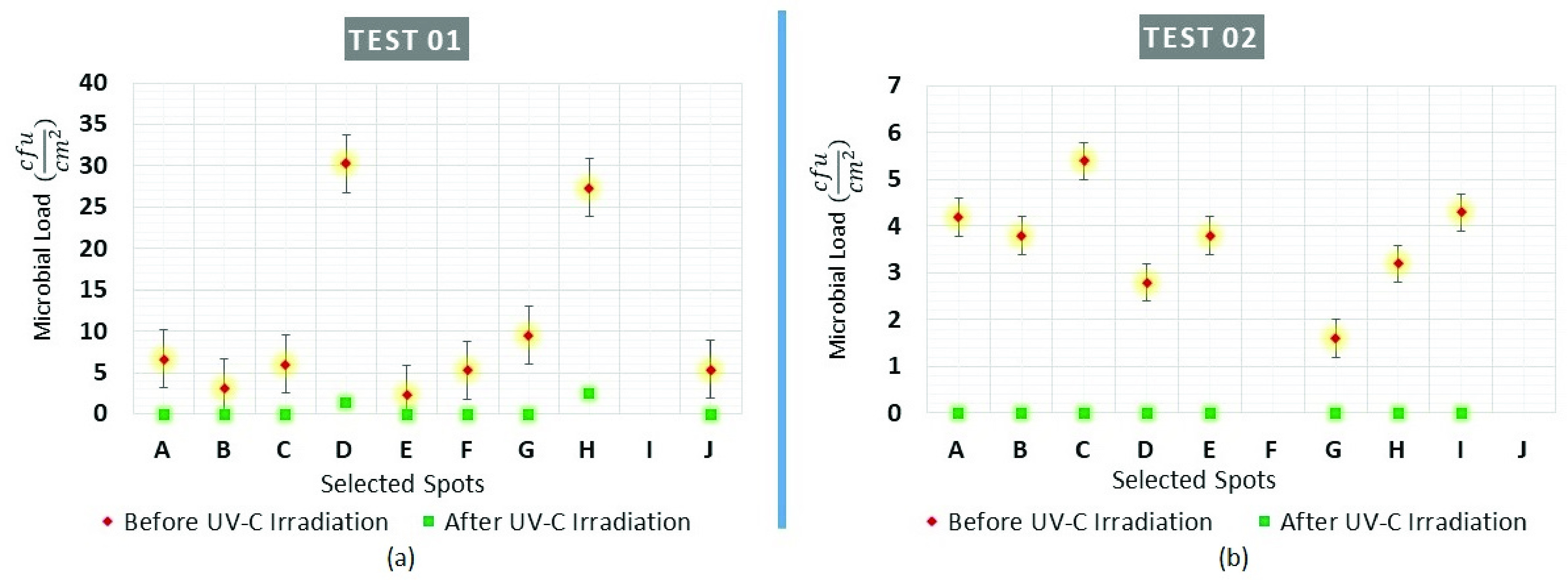
Status of microbial load (a) before the irradiation (Pre-UV) (b) after the irradiation (Post-UV).

**FIGURE 8.**
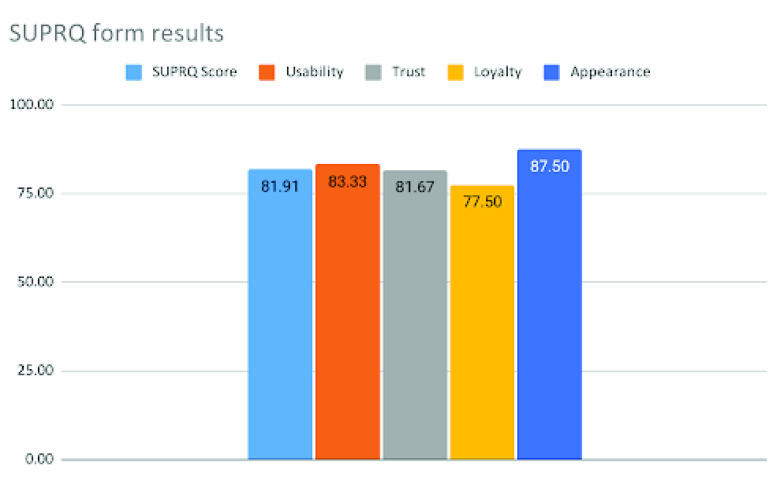
SUPR-Q results.

**FIGURE 9.**
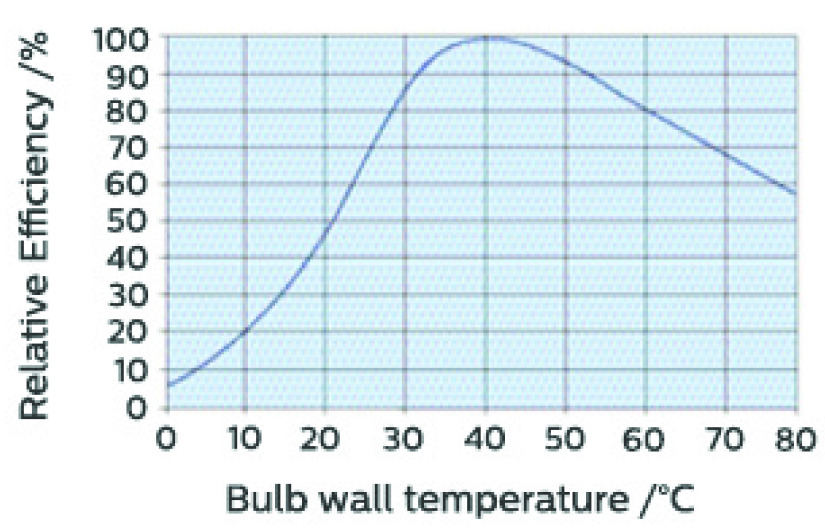
Temperature effect on relative efficiency of UVC lamp (PHILIPS, 2021).

**FIGURE 10.**
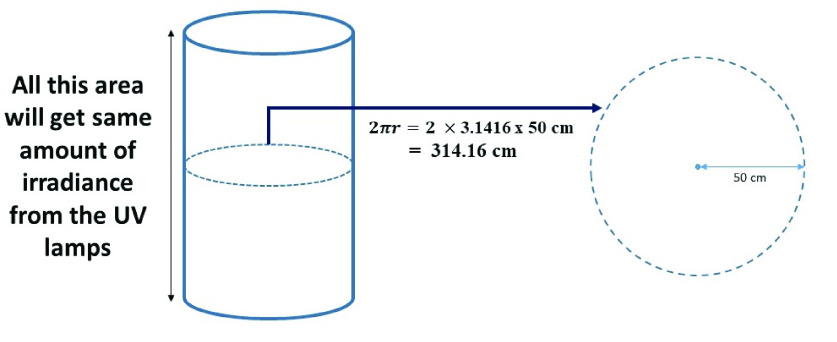
Distribution of irradiation around the robot.

**FIGURE 11.**
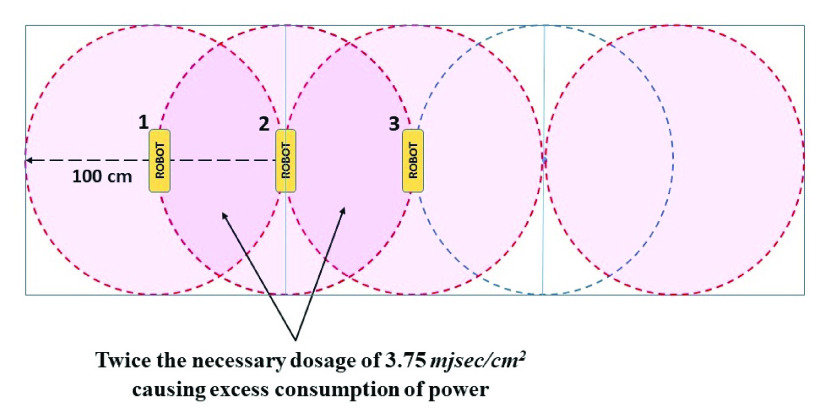
Traversal of UVC-PURGE-Overlapped regions.

**FIGURE 12.**
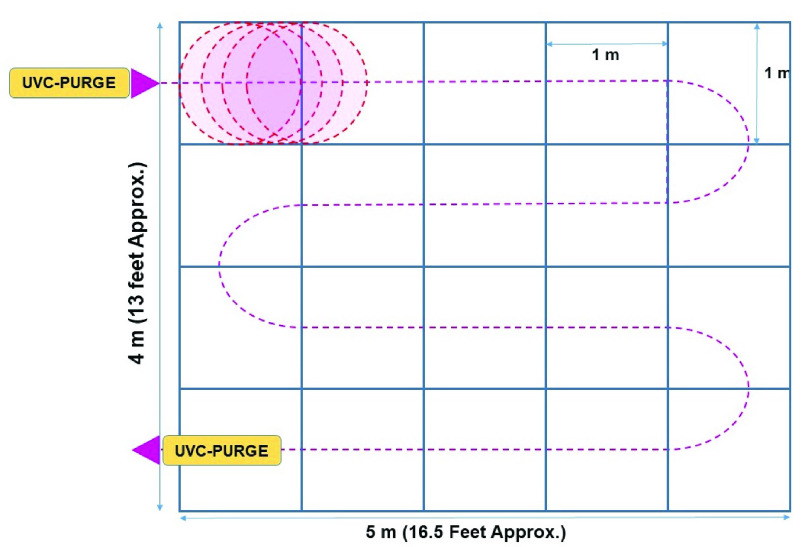
Schematic diagram of the disinfection procedure of a standard room using UVC-PURGE.

### C. System Components and Integration

The system architecture of the robot has been shown in Figure 3. Raspberry pi 3B+ has been used as the master processing unit of this robotic system. The robot has a four-wheel-drive mechanism with motor drivers (BTS7960). Pi-camera with resolution 720p has been mounted at the front of the robot and has been connected with the master processor to provide real-time camera feedback. While navigating, this semi-autonomous robot is capable enough to detect any obstacles and protect itself by detecting them. The Robot Operating System (ROS) has been used to design the navigation modes of the robot and for inter-process communication. Arduino microcontroller has been used as the slave-processing unit for accurate obstacle detection mechanisms through six ultrasonic sensors (HC-SR04) and for system monitoring using internal voltage, current, and temperature sensors. A customized WiFi system has been integrated within the robot that has a coverage area of up to 1600sq feet. Additionally, the robot has been powered using a 12V Lead-acid battery capable of generating 360 Watt/hour. This power subsystem provides a battery backup of 1.5 hours and needs 4 hours for full charging. The expected work cycle of the robot is 2–3 years and the expected lifetime of the UVC lamp is 8,000 to 10,000 hours.

### D. System Software and Mobile Application

A mobile application (android) (Figure 4) has been developed for controlling the robot wirelessly. The application shows video feedback and system information and sends command information from the user to the robot. The maximum value (100%) in the speed bar of the application allows the user to traverse 1 meter in 5.19 seconds which will ensure the rational lethal dose ( $3.75~mJ sec/cm^{2}$) for SARS-CoV-2. Additionally, a reverse camera line has been provided for drive assistance to the users. With similar features, a web-based dashboard has been developed for cross-platform compatibility to enable organizational use such as office or hospital. Being wirelessly controlled by mobile application or web-based dashboard, UVC-PURGE is very user-friendly with 1600 square feet coverage area. Figure 5 shows the basic workflow of the system. Two modes have been provided in the mobile application as *Normal* and *Protected* to allow the users to navigate in congested spaces. In the Protected mode, the robot automatically detects obstacles and protects itself by stopping at a 0.5 m safety distance. However, this is not very ideal for navigating the robot in a congested space. Thus, in the Normal mode, the user has the flexibility to navigate it freely reducing the safety distance to 0.15 m.

The system needs to be powered on using the Master Switch. Yellow Light in Status indicators will be flashed within 30–40 sec to confirm that the system is ready to be connected. In this state, the robot works as a hotspot for the users and the user can connect the mobile application or web-based dashboard with the robot through proper authentication. Two separate servers running in the Raspberry Pi ensure video transmission using User Datagram Protocol (UDP) and command transmission using Transmission Control Protocol (TCP) simultaneously. TCP ensures the data reliability for command and control between the robot and the dashboard and UDP ensures the real-time video transmission. Moreover, the average response time to transmit a command and the robot to respond is approximately 0.0045 seconds for mobile application commands and 0.0091 seconds for keyboard commands using the web-based dashboard. The robot has two layers of safety measures. Continuous acknowledgment packet transmission in TCP ensures that the robot will automatically turn off in case of communication failure. Additionally, the user will also receive the low battery notification at 20% of battery health. The Master Switch ensures timely shutdown of the whole system in extreme emergencies.

## V. Evaluation of the Robot

Evaluation of UVC-PURGE was undertaken to validate the performance of the developed robotic system. The evaluation study was designed in two phases to evaluate both the effectiveness and usability of the robotic system. The first phase was designed and conducted to validate the effectiveness of UVC-PURGE to inactivate the SARS-CoV-2 virus within the estimated disinfection time and the feasibility of semi-autonomous navigation. The test was being conducted with the help of one of the leading hospitals of Bangladesh consisting of 250+ patient capacity in Dhaka, Bangladesh. The second phase was to evaluate the usability of the UVC-PURGE system by the general people especially the maintenance staff using a hybrid approach of SUPR-Q forms and subjective evaluation. This was conducted with the help of one of the telecommunication monitoring companies located in Dhaka, Bangladesh who was the pilot user of the developed robot. This time, users from different location were selected to include more persons from different places in testing the system.

### A. Effectiveness

#### 1) Sample Preparation

Surfaces chosen to be sampled were having a high probability of human contact. The samples were collected by the same lab technician throughout the study. At first, the surface to be used as a sample was divided into eight $1\times 1$ cm squares for the preparation of the swabs. The swabs were moistened by Phosphate Buffered Saline (PBS) [1% glycerol solution (GS)]. The swabs were then used to collect a sample from four alternate squares before the UVC treatment of the developed robot. After the collection, sample swabs were immersed in ml PBS and refrigerated. After the UV irradiation of UVC–PURGE, surfaces were re-sampled using the remaining four squares at each sample location following the same pre-treatment phase. The collected samples before-irradiation and after-irradiation were diluted in 1:10 PBS. Than 150 $\mu \text{l}$ of it was plated onto nutrient agar with four technical replicates and incubated at $37^\circ \text{C}$ to grow statically for 48 hours. Following the incubation, colony-forming units per square centimeter ( $cfu/cm^{2}$) were used as the unit of measurement for the documentation of the microbial load.

#### 2) Test Procedure

One of the operation theatres (Room-1) of size 24 $m^{2}$ (6 m $\times $ 4 m) and a 5-bed patients’ ward (Room-2) of size 40 $m^{2}$ (8 m $\times $ 5 m) were chosen to be the evaluation rooms. In the operation theatre area, 10 surfaces were sampled (door handle, switchboard, tabletop of two tables, two bedside handles, bedside table, the armrest of a chair, x-ray viewer, shelf handle) and labeled as 1A-1J (see Appendix B: Table 3). Similarly, in the 5-bed patients’ ward area, another 10 surfaces were sampled (door handle, switchboard, two bed handles, window countertop, armrest of two chairs, tabletop, shelf handle, TV shelf top) and labeled as 2A-2J (see Appendix B: Table 4). The labeled surfaces of Room-1 and Room-2 are illustrated on the schematic diagram in Figure 6a and Figure 6b respectively. The tests were conducted once in each of the rooms using two different simulated obstacle setups and have been named Test-1 (Room-1) and Test-2 (Room-2). During the evaluation study, humans were not allowed to enter the room and two of the maintenance staff of the hospital controlled the robot from outside the room using the developed mobile application. Routes followed by the users to navigate the robot in Test-1 and Test-2 have been illustrated in Figure 6a and Figure 6b respectively. In Test-1, five obstacles were placed in the route to create a narrow space. The creation of the narrow space (Figure 6a) forces the user to switch off the “Protected” mode to traverse through the space. In Test-2, the robot was driven in “Protected” mode throughout the room and three obstacles were placed in the route as marked in the Figure 6b. In Test-1 the robot was controlled at a speed of a minimum of 40% to a maximum of 55% speed. The presence of both Gram-positive and Gram-negative bacteria was detected in the samples before the treatment of UVC irradiation. Levels of microbial load ( $cfu/cm^{2}$) in each surface were measured both before and after the UVC treatment and the achieved UVC doses corresponding to each sampling surface are presented in the supplemental material (see Appendix B: Table 3 and 4).

**TABLE 3:** Reductions in Colony-Forming Unit (CFU) per Square Centimetre ( $cfu/cm^{2}$) in the Operation Theatre (Room-1) Area After the Use of the UVC-PURGE (Including the Speed of the Robot and UVC Dose Received)

Label of the Surface	Sampling area	Time of Sampling	Number of samples	CFU per square Centimetre $(cfu/cm^{2})$	Speed of the Robot	UVC Dose $(mJ sec/cm^{2})$
1A	Door Handle	Before UVC Irradiation	4	4.7	50%	7.5
		After UVC Irradiation	4	0		
1B	Switch Board	Before UVC Irradiation	4	3.2		
		After UVC Irradiation	4	0		
1C	Table top 1	Before UVC Irradiation	4	6		
		After UVC Irradiation	4	0		
1D	Bedside handle 1	Before UVC Irradiation	4	30		
		After UVC Irradiation	4	1.4		
1E	Arm rest of Chair	Before UVC Irradiation	4	2.4	40%	9.375
		After UVC Irradiation	4	0		
1F	Table top 2	Before UVC Irradiation	4	6		
		After UVC Irradiation	4	0		
1G	Bedside Table	Before UVC Irradiation	4	9.5	55%	6.82
		After UVC Irradiation	4	0		
1H	Bedside handle 2	Before UVC Irradiation	4	27.3		
		After UVC Irradiation	4	2.5		
1I	X-ray viewer	Before UVC Irradiation	4	0		
		After UVC Irradiation	4	0		
1J	Shelf Handle	Before UVC Irradiation	4	5.4		
		After UVC Irradiation	4	0

**TABLE 4:** Reductions in Colony-Forming Unit (CFU) per Square Centimetre ( $cfu/cm^{2}$) in the 5-Bed Patients’ Ward (Room-2) After the Use of the UVC-PURGE (Including the Speed of the Robot and UVC Dose Received)

Label of the Surface	Sampling area	Time of Sampling	Number of samples	CFU per square Centimetre $(cfu/cm^{2})$	Speed of the Robot	UVC Dose $(mJ sec/cm^{2})$
2A	Door Handle	Before UVC Irradiation	4	4.2	55%	6.82
		After UVC Irradiation	4	0		
2B	Switch board	Before UVC Irradiation	4	3.8		
		After UVC Irradiation	4	0		
2C	Bed 4 handle	Before UVC Irradiation	4	5.4		
		After UVC Irradiation	4	0		
2D	Table top	Before UVC Irradiation	4	2.8	40%	9.375
		After UVC Irradiation	4	0		
2E	Arm rest of chair 1	Before UVC Irradiation	4	3.4	50%	7.5
		After UVC Irradiation	4	0		
2F	Window coutertop	Before UVC Irradiation	4	0		
		After UVC Irradiation	4	0		
2G	Arm rest of chair 2	Before UVC Irradiation	4	1.6		
		After UVC Irradiation	4	0		
2H	Bed 1 handle	Before UVC Irradiation	4	3.2	55%	6.82
		After UVC Irradiation	4	0		
2I	Shelf handle	Before UVC Irradiation	4	4.3		
		After UVC Irradiation	4	0		
2J	TV Shelf top	Before UVC Irradiation	4	0		
		After UVC Irradiation	4	0		

#### 3) Results

Results from the swab testing indicate that UVC-PURGE was successful in eliminating the measurable microbial load from the given samples. Both Gram-positive and Gram-Negative bacteria were detected with different measurements of bacterial colonies. There was a presence of bacteria in 09 out of 10 surfaces (except 1I) before the irradiation in Test-01 and 08 out of 10 (except 2F and 2J) surfaces in Test 02. The results after the disinfection by the robot as shown in Figure 7 showed that UV irradiation was efficient enough to reduce the level of bacterial load ( $cfu/cm^{2}$) in most of the surfaces. UVC-PURGE was capable of inactivating 100% of the bacterial colony in 07 out of 09 infected surfaces in Test-01 and 08 out of 08 contaminated surfaces in Test-02. In Test-1 at surface ‘1D’ and ‘1H’ (Bedside Handles), a relatively high concentration of bacterial load of *Staphylococcus aureus* (Gram-Positive), *Staphylococcus epidermidis* (Gram-Positive), and *Staphylococcus saprophyticus* (Gram-Negative) were observed before the irradiation. After the UV irradiation of the UVC-PURGE, the bacterial load of these surfaces wasn’t fully eliminated. However, 95.33% and 90.9% of the bacterial load was eliminated in the case of surface 1D and surface 1H respectively, which shows a significant reduction. Additionally, in both the tests, the robot was able to detect all the obstacles placed in the route and maintained the safety distance according to the selected mode of operation. The user was successful to control the robot in the narrow space at Test-1 using the developed mobile application without the help of the research team.

### B. Usability Evaluation

In order to determine the usability of the system from the perspective of target users, a study in collaboration with a team of maintenance staff and engineers of one of the reputed telecom companies of Bangladesh was conducted.

#### 1) Study Procedure

The maintenance team was provided with the UVC-PURGE prototype and the mobile application. A training session was conducted for them which lasted for about 15 minutes. During the training session, a small brief was provided regarding how UVC rays can be utilized for disinfection purposes. Then an overview of the prototype was given, during which different components of the robotic system were described including the positioning of the wheels, the location of the Master Switch, indicators, UI of the mobile application, etc. Furthermore different precautionary measures that are to be taken while using the UVC-PURGE prototype were mentioned. The precautionary measures included controlling the robot from a safe distance preferably from a neighboring room, avoiding placing any weights on the top of the prototype, etc. After that, a tutorial for the mobile application was imparted. During the tutorial, different components of the UI of the app were described including the Protected and Normal mode switch, the four directional buttons, the stop button, the speed adjustment seekbar, the camera view, and the about button.

After concluding the training sessions, The first of the three UVC-PURGE robots were delivered to the testing team for use in their routine disinfection procedure. It was agreed that for the next 1 week, the robot would be used for disinfecting 4 different rooms every day which included the conference room, the dining room, the employee washroom, and the Network Operation Control (NOC) room. 13 maintenance staff and 2 electrical engineers were tasked with testing the prototype in the above-mentioned rooms at different times during their working hours. Upon the initial pilot testing, it was found that about on average of 45 minutes were needed for disinfecting all of the mentioned rooms with a range of a minimum of 32 minutes to a maximum of 76 minutes. However, the average of last week(36 minutes approximately) was less than the average time for the first week (57 minutes approximately). The manual time required for 4 cleaners to disinfect the same number of rooms was 1 hour and 25 minutes. The dining hall was the largest room and it required maximum time (12 minutes) on average for disinfection. Again, the NOC room was comparatively a more congested room with various furniture where the prototype required navigation using the Normal mode.

After the completion of the 7 days testing period, an evaluation form was supplied to all 15 users. The evaluation form was a Standardized User Experience Percentile Rank-Questionnaire (SUPR-Q) [35] form for evaluating the four aspects related to the robot: Usability, credibility (trust), appearance, and loyalty. In the SUPR-Q form, the following questions were placed:

1. The product is easy to use.
2. It is easy to navigate using the mobile application.
3. I feel comfortable purchasing the robot.
4. I feel confident conducting the disinfection using the product.
5. I will reuse this product in the future.
6. I find the robot and the mobile app to be attractive.
7. The product has a simple presentation.
8. How likely are you to recommend this product to a colleague and others?

In the questionnaire set, questions 1 and 2 have been used for determining the usability of the system. Questions 3 and 4 have been used for determining the credibility of the system to the users. Questions 5 and 8 (Net Promoter Score Question) have been used to determine whether the users are loyal to the system and how much likely are they going to recommend the system to others. Finally, questions 6 and 7 have been used to determine whether the users like the appearance of the system or not. Responses regarding questions 1 to 7 had been taken using the Likert scale of [55] 5, while the response of question 8 was obtained on the scale of 10.

After receiving the responses from the users through the SUPR-Q form, feedback and opinions from the users were obtained through a semi-structured interview for subjective evaluation. The audio of the interview sessions was recorded after taking their permission. The audio clips were transcribed later on into texts and thematic analysis was conducted on the collected texts.

#### 2) Study Findings

The results obtained from the SUPR-Q forms show that the average SUPR-Q score expressed in percentage for the system is 81.91%, which implies that its different features have above-average qualities. The percentage of average Usability value from the forms was found to be 83.33%. Since it is above 50%, hence it has been found by the users to be highly usable. Similarly, the percentage of average Trust value has been found to be 81.67% which means that the users trusted the system’s capabilities to carry out its intended tasks. The percentage of average Loyalty value has been found to be 77.50%. Though this value is comparatively less than the other values, it is still way above 50% and hence it means that the users are more likely to recommend the prototype to other potential users. Finally, the percentage of average Appearance value has been found to be 87.50% which means that the users liked the appearance and design of the system. The results obtained from different users using the SUPR-Q form are depicted in the graph in Figure 8.

Seven resultant themes were obtained from the responses received from the semi-structured interviews through thematic analysis. The themes that emerged from the analysis reflected the thoughts of the participants regarding the system. The themes are briefly described below.

##### a: Requiring Minimal Training

It was identified from the semi-structured interviews that the prototype and the mobile app were found to be very intuitive to use for all of the users. The entire system was found by the users to be free from unnecessary complexities. Regarding this one of the electrical engineers said, “Not many electrical devices are so simple to use. In fact, this device is performing a very serious and to some extent life-threatening task. But despite such serious contexts, it is really appreciable that the robot can be controlled and used by anyone who has very little technical experience and education.” Regarding the training sessions that were conducted earlier before handing over the three prototypes, one of the maintenance staff said, “During the training session, it was very easy for us to understand how the robot works. Initially, we were a bit overwhelmed thinking that controlling a robot would be a very challenging task for us. But later we found that it is very simple and easy to use.”

##### b: Reduced Fear of Contamination and a Helping Aid

Previously, while manually disinfecting the surfaces, the cleaning and maintenance staff had to stay in close proximity to the potentially contaminated surfaces. However, upon the inclusion of the developed robot into the disinfection procedure, the user’s fear of being contaminated and becoming sick greatly reduced. Moreover, the robot helped them to perform disinfection with ease and comfort. Hence, initially, though some of the workers questioned us about whether this system might pose threats to their job, later on upon asking all of the cleaners expressed their acceptance of the system.

##### c: Ease of Disinfection

Another theme identified from the thematic analysis was the ease of disinfection. Previously, while disinfecting different rooms, the maintenance staff had to spend a lot of time preparing disinfection mixtures, applying the mixtures on different furniture in a room and on the floor, storing the disinfection chemicals, etc. Furthermore, the corrosive nature of disinfecting chemicals is another harmful aspect of traditional disinfecting procedures. But with the introduction of the UVC-PURGE prototype, these problems can be easily avoided. Regarding this one of the maintenance engineers said the following: “Previously, disinfecting a room was a very lengthy task for us, we had to bring out the disinfectants and cleaning tools like mops and rags. Then we had to prepare the cleaning mixture with water and chemicals. Then we had to physically apply the disinfecting mixtures in different places in a room. Finally, we had to store the chemicals with a lot of effort. But using the robot requires none of those tasks and hence I find that our work has become much easier comparatively.” One of the main factors that resulted in ease of disinfecting was the semi-autonomous mode of the prototype. About this, one of the maintenance staff had the following opinion: “After letting the robot move in our dining hall, we were able to sit and relax while the robot was doing the cleaning. From time to time we just changed the direction of the movement of the robot, other than that, we did not have to do anything else.”

##### d: Reducing Fear of Contamination

From the interviews, it was found out that using the UVC-PURGE prototype resulted in reducing fear of being infected by the Corona Virus. The cleaning and maintenance staff felt comfortable and safe as the prototype facilitated disinfecting from a distance. Regarding this one of the cleaning staff said the following: “Previously, while carrying out the disinfecting tasks, I used to feel scared. I always had a feeling that I was going to become contaminated by the virus and I may even carry the virus to my home and infect my family members. But this robot helps me to stay relaxed to some extent.”

##### e: Unable to Run on Mobile Devices That Does Not Support the Android OS

A limitation that was obtained from the participants was that the users of Non-Android Smartphones were unable to run the mobile app and hence were unable to control the robot. The developed mobile app was suitable for only Android-based smartphones. But because of this, users of other mobile OS were not able to install it. Regarding this one of the electrical engineers said the following, “I use iPhone 10, hence it was not possible for me to install the app on my mobile. I had been doing the testing using the web-based dashboard. However, I will prefer using it as a mobile application” The other electrical engineer made some suggestions regarding the app development, “You can make another app targeting the iOS platform as well as other platforms. For this, you can use some cross-platform development tools. I would suggest Flutter for this. Also, you can use iOS-based development tools as well, for example, Swift.”

##### f: Ease of Navigating in Congested Areas

One of the positive aspects identified from the prototype during testing was the ease of navigation while disinfecting the congested areas. The NOC room was comparatively more congested with several pieces of furniture and telecommunication equipment. Initially, the users thought that controlling the robot in such a congested environment would result in difficulties. However, due to the presence of ultrasonic sensors, such problems were easily avoided. The ultrasonic sensors are able to detect both vertical and horizontal obstacles at 0.5 meters and 0.15-meter distances in protected and normal mode respectively. Furthermore, the presence of the live video feed and the intuitive control buttons also enabled the users to effectively navigate the system. Regarding this one of the cleaning and maintenance staff said the following: “Disinfecting the NOC room had always been a tedious task for us. It is equipped with so many pieces of furniture that are required to be cleaned and disinfected regularly. However, we found that disinfecting that place with your robot made the task very much easier.” Regarding this feature, one of the electrical engineers said the following: “It was indeed a brilliant idea to use the ultrasonic sensors in this prototype of yours.”

##### g: Unable to Store Predefined Movement Paths

Another limitation was pointed out by the engineers which were regarding defining movement paths for the robot. One of the engineers said the following regarding this, “Every day while testing we had to provide the same movement commands. Though the robot is semi-autonomous and can be left unattended for a long time, it would be better if we could store the movement paths of the robots. In that way, every day, the robot will only have to be placed at a specific point in the room and it will repeat the recorded movements on its own without external intervention”. Regarding, one of the cleaning and maintenance staff said the following, “If your robot was fully automated as sir (the electrical engineer) said, then we could leave a robot in a room and go to another place to do some other tasks. It would really help us to save our time and complete more tasks.”

## VI. Discussion and Conclusion

The successful evaluation study shows that the developed system is effective in case of neutralizing the bacterial load. Pre-treatment swab testing revealed the presence of a variety of microbial loads of both Gram-positive and Gram-Negative bacteria (*S. epidermidis*, *S. aureus* and *S. saprophyticus*) at 17 out of 20 test surfaces in the conducted tests. After the treatment of the UVC irradiation of the robot, the microbial load was detected in only 2 (1D and 1H surfaces in Test-1) out of 17 test surfaces where the greatest concentration of microbial load of 30 (1D) and 27.3 (1H) $cfu/cm^{2}$ was observed (Figure 8). However, UVC-PURGE could reduce the microbial load significantly (95.33% in ‘1D’ and 90.9% in ‘1H’) in both tests. We could not evaluate the robot on the SARS-CoV-2 due to the current unstable nature of the virus and local legal restrictions on virus culture. However, the performance of the robot in the case of inactivating coronavirus can be estimated from the generated output of the conducted tests. Recent studies [15]–[21] stated that the rational lethal dose for inactivation of coronavirus is 3.75 $mJ sec/cm^{2}$. During the disinfection process of the evaluation study, the robot was controlled within a range of 40% to 55% of its maximum speed, which has ensured irradiation of 9.375 $mJ sec/cm^{2}$ to $6.82~mJ sec/cm^{2}$ on the sample surfaces. The findings of the evaluation study showed that the robot was successful(Figure 8) at inactivating *S. aureus* that requires an inactivation UVC dose of $6.06~mJ sec/cm^{2}$ [56], which is much higher than the rational inactivation dose of SARS-CoV-2 ( $3.75~mJ sec/cm^{2}$). The robot has successfully inactivated the bacterial load which is higher resisting than SARS-CoV-2 which indicates the expected success of the robot in the case of SARS-CoV-2 (coronavirus). The robot has also demonstrated its semi-autonomous capability by successfully detecting all the obstacles during the conducted evaluation study. Additionally, its small form factor along with real-time video feedback through the mobile application has helped the user to manoeuvre the robot in the simulated narrow passage.

From the usability evaluation of the system, it was found that the robot had achieved promising results from SUPR-Q-based evaluations. Each of the values obtained from the SUPR-Q-based evaluation was way above average, which further reinforced the fact that the robot has been appreciated and accepted by the users. Furthermore, the thematic analysis conducted for testing the usability of the system showed that the robot is operable by users with minimal training, capable of easily disinfecting probably contaminated areas, capable of providing mental ease to the cleaning staff, and carries out disinfection in congested areas. Though the thematic analysis showed that the robot can be further enhanced by developing a platform-independent mobile app and path remembering capabilities, the number of positive themes that emerged from the thematic analysis is twice the number of negative themes.

The static UVC Disinfection method can be effective on surfaces where light can be directly irradiated. However, the shaded areas or the areas which are not under the arcs of the UVC light can remain contaminated. This problem can be solved using a semi-autonomous robotic system by navigating it effectively through all the corners of the infected area. This task can also be achieved using a fully autonomous robot. However, it will be difficult for an autonomous robot to navigate in a congested place with narrow pathways as the navigation will be based on the obstacle avoidance procedure using sophisticated vision technology (such as LIDAR or Depth sensing camera). Providing the robotic system with such highly precise sophisticated vision technology is an expensive task that will increase the total production cost of a robot. Again, compromising with the quality of the sensor reduces the precision of path planning and thereby will not be useful while navigating in a congested narrow space. For addressing this issue, a user-friendly control dashboard with real-time camera feedback and a *Normal* mode of operation with a reduced obstacle detection capability is installed in the developed UVC-PURGE for enabling the user to drive the robot in such situations. The user can navigate in narrow spaces easily without the fear of physical collision of the robot by choosing the *Normal* mode of operation and using the real-time camera feedback of the UVC-PURGE. Thus, the developed semi-autonomous robot can provide similar services to the prevailing autonomous disinfection robots with a lower production cost. However, there are some specific regions, for example, the backside of the door handle, which will remain shaded with maximum effort. These regions have to be disinfected using a manual wipe or conventional method. Nevertheless, the effort and time of wiping those small areas will be significantly reduced in comparison to conventional disinfection methods as shown in the evaluation study. The developed system disinfected a room with the dimension of 4m $\times 5\text{m}$ in less than 5 minutes (4 minutes 51 seconds) whereas the conventional disinfection method required an average of more than 17 minutes (17.65) and also depends on the efficiency of the maintenance staff. Thus, UVC-PURGE can disinfect 3 times more area than the conventional disinfection methods in a similar timeline.

Moreover, Validation of the effectiveness in lab environment is completed for the developed system which ensures a technology readiness level of 4 (TRL-4). It is evident from the findings of the market analysis that UVC-PURGE with a market price of less than 800 USD is cost-effective in comparison with the average price of 55,165 USD (Table 1) of the prevailing UV robots. Thus, the developed robot is almost five times more cost-effective than any other similar product in the market. To the best of the authors’ knowledge and from the findings of the market analysis, the developed robotic system is likely to be the most cost-effective product with a price of less than 800 USD only. Though the robot is not fully autonomous i.e. requires a human controller to drive the robot, it can serve the purpose of ensuring safe, time-efficient, and cost-effective disinfection. Moreover, the advantage of live video streaming capability through a user-friendly dashboard (mobile/web-based application) along with limited cross-platform compatibility is also a unique feature compared with the other existing robotic systems. Additionally, unlike a few of the existing robots, Dual Modes of Operation assisted by the lightweight and small form factored body structure helps UVC-PURGE to move easily in congested spaces.

This robot is applicable for deploying in any indoor environment which includes the following use cases: a) maintenance staffs in medical facilities using it to disinfect COVID patient ward, ICU, operation theatre, and other compartments; b) business personnel using it to disinfect workspace; c) maintenance staffs using it to disinfect classrooms, laboratories, etc. in an educational/research institution and d) common people using it for day to day disinfection of personal apartments. Moreover, in the post-pandemic world, IPC will remain an important matter of concern, especially for medical facilities and biomedical research facilities. Additionally, recent studies [36], [37] have shown that the residual effect of SARS-CoV-2 (coronavirus) may continue to persist for a very long time and in the future, it might become endemic. Thus, this cost-effective disinfection robot can be an extremely useful IPC tool for day-to-day life in the post-pandemic world.

The system can be further improved by addressing the negative themes (Unable to store predefined movement paths and Unable to run on mobile devices that do not support the Android OS) that emerged from the usability evaluation study. The inclusion of the feature of accepting predefined routes as input from the users can improve the usability of the robot. Furthermore, the developed robot will be more extensive if the mobile application is developed with cross-platform capability. Though this research has mainly focused on surface disinfection using a semi-autonomous robotic platform, however, UVC-PURGE will likely be effective in the case of air purification as well. This can be an interesting future research scope of the developed robotic system. Moreover, validation of the structural efficiency of the developed robot can help to improve the performance of the system in the future. Additionally, the developed system is ensuring the minimum power consumption by ensuring a calculated maximum speed (see Appendix A). The developed robot is currently a semi-autonomous system. Improving the autonomy of the robot while maintaining an affordable cost can also be an important future research scope.

## Appendix A Calculation of Disinfection Time of UVC-Purge

Stefan Boltzmann (S-B) Law, $J_{r}=\in \delta T^{4}$ [57]

Where, $J_{r} =$ Radiation Flux, $\in $, Emissivity = 0.82 (254 nm) $\delta $, S-B Constant = 5.6697 x 10- $8\,\,Wm^{-2}K^{-4}$

T, Temperature of the UVC Lamp

\begin{align*} \text {T}1=&322~\text {K}\\ \text {T}2=&321.4~\text {K}\\ \text {T}3=&323.9~\text {K}\\ \text {T}4=&322.5~\text {K}\\ \text {T}5=&323.1~\text {K}\\ \text {T}6=&322.8~\text {K}\end{align*}

Total Radiation Flux,

\begin{align*} J_{r}=&\sum \nolimits _{i=6}(\text {J}_{\text {i}})\\=&(499.8 \!+\! 497.32 + 511.70 \!+\! 502.91 \!+\! 506.67 \!+\! 504.79)\\=&3023.19~W\end{align*}

At $50^\circ \text{C}$, due to the temperature affect the efficiency of the output of the UVC lamps is 92% [58] as shown in Figure 9

Thus, Updated Jr = ( $3023.19\times0.92$) = 2781.33 W

Vertical distance from the target, a = 50 cm

Length of the Lamp, l = 78 cm

Now, While a < 0.51

Irradiance,

\begin{align*} \text {E}=&J_{r}/2\Pi ~\text {al}~[{58}]\\=&2781.332 \times 3.1416 \times 50 \times 78\\=&0.11350~J/cm^{2}\\=&113.50 ~mJ sec/cm^{2}\end{align*}

At once the overall surface is receiving the same amount of radiation as shown in Figure 10

Rational Lethal Dosage (D90-D99.9) inactivation $=3.75\,\,mJ sec/cm^{2}$

Time required cleaning 1 m diameter surface = (314.16 cm $\times 3.75\,\,mJ sec/cm^{2}$) $\div $ ( $113.50~mJ/cm^{2}$) = 10.380 sec

However, as shown in Figure 11 if the speed is set for the robot to traverse 1 meter in 10.380 sec the surface area will get twice the required lethal dosage, which will cause unnecessary excess consumption of power.

Optimization of power consumption can be achieved through 1/2 of the calculated time. Thus, 12 of 10.380 sec = 5.19 sec is required for disinfecting a 1 m diameter surface.

Therefore, Maximum Speed of UVC-Purge = 1 m in 5.19 sec = 0.193 m/sec

=19.3 cm/sec

In Figure 12, a schematic diagram of the disinfection procedure of a standard room (13 feet 16.5 feet) using UVC-PURGE has been shown. There is a total ( $4\times 5$) of 20 general blocks, 3 blocks to take turns. At the maximum speed of UVC-PURGE, the blocks can be disinfected in 119.37 seconds. Thus, the disinfection procedure will take 119.37 sec i.e. 2.5 minutes approximately provided there is no obstacle in the room. However, in the case of a furnished room, this may be increased to 4–5 minutes.

*Summary:*

1) UVC-PURGE can provide disinfection of 1 square meter surface by traversing at 1 meter in 5.19 seconds.
2) Maximum Speed of UVC-PURGE has been set to 19.3 cm/seconds or 0.193 m/sec.
3) The robot is capable of disinfecting a standard bedroom of (4 m $\times $ 5 m) with furniture within 4–5 minutes.

## Appendix B Evaluation Score Documentation

See Tables 3 and 4.
